# Mutant TDP-43 and FUS Cause Age-Dependent Paralysis and Neurodegeneration in *C. elegans*

**DOI:** 10.1371/journal.pone.0031321

**Published:** 2012-02-21

**Authors:** Alexandra Vaccaro, Arnaud Tauffenberger, Dina Aggad, Guy Rouleau, Pierre Drapeau, J. Alex Parker

**Affiliations:** 1 The Research Centre of the University of Montreal Hospital Centre, Université de Montréal, Montréal, Québec, Canada; 2 Centre of Excellence in Neuromics, Université de Montréal, Montréal, Québec, Canada; 3 Département de Pathologie et Biologie Cellulaire, Université de Montréal, Montréal, Québec, Canada; 4 Département de Médecine, Université de Montréal, Montréal, Québec, Canada; Mayo Clinic, United States of America

## Abstract

Mutations in the DNA/RNA binding proteins TDP-43 and FUS are associated with Amyotrophic Lateral Sclerosis and Frontotemporal Lobar Degeneration. Intracellular accumulations of wild type TDP-43 and FUS are observed in a growing number of late-onset diseases suggesting that TDP-43 and FUS proteinopathies may contribute to multiple neurodegenerative diseases. To better understand the mechanisms of TDP-43 and FUS toxicity we have created transgenic *Caenorhabditis elegans* strains that express full-length, untagged human TDP-43 and FUS in the worm's GABAergic motor neurons. Transgenic worms expressing mutant TDP-43 and FUS display adult-onset, age-dependent loss of motility, progressive paralysis and neuronal degeneration that is distinct from wild type alleles. Additionally, mutant TDP-43 and FUS proteins are highly insoluble while wild type proteins remain soluble suggesting that protein misfolding may contribute to toxicity. Populations of mutant TDP-43 and FUS transgenics grown on solid media become paralyzed over 7 to 12 days. We have developed a liquid culture assay where the paralysis phenotype evolves over several hours. We introduce *C. elegans* transgenics for mutant TDP-43 and FUS motor neuron toxicity that may be used for rapid genetic and pharmacological suppressor screening.

## Introduction

Amyotrophic Lateral Sclerosis (ALS) is a late-onset progressive disease affecting motor neurons ultimately causing fatal paralysis [1], [2]. Most cases are sporadic, but ∼10% of patients have an inherited familial form of the disease. Dominant mutations in SOD1 (copper/zinc superoxide dismutase 1) account for ∼20% of familial ALS cases and ∼1% of sporadic cases [1]. The recent discovery of mutations in TAR DNA-binding protein-43 (TDP-43) and Fused in sarcoma (FUS, also named TLS) in both familial ALS and frontotemporal dementia (FTD) has shifted research into disease mechanisms and potential therapeutics [3]–[9].

TDP-43 and FUS are evolutionarily conserved DNA/RNA binding proteins that shuttle between the nucleus and cytoplasm having multiple roles including DNA transcription and RNA processing [3], [9]–[12]. Mutant TDP-43 and FUS (mTDP-43 and mFUS) are found in cytoplasmic inclusions in the disease state while the accumulation of wild type TDP-43 and FUS (wtTDP-43 and wtFUS) are observed in an increasing number of disorders including Alzheimer's Disease, Parkinson's Disease and the polyglutamine diseases (reviewed in [10]). The pathogenic mechanisms for mutant TDP-43 and FUS age-dependent neuronal toxicity remain unclear. As of now there is no consensus whether mutant TDP-43 and FUS employ a loss-of-function, a gain-of-function, or both in motor neuron cell death.

Since TDP-43 and FUS are evolutionarily conserved we used the nematode *Caenorhabditis elegans* to investigate mutant TDP-43 and FUS age-dependent neurodegeneration. We created transgenic nematodes that express full-length wild type or mutant TDP-43 and FUS in the worm's GABAergic motor neurons. Transgenic TDP-43 and FUS worms recapitulate a salient feature of ALS; they display adult-onset, age-dependent, progressive paralysis and degeneration of motor neurons. Importantly, mTDP-43 and mFUS, but not wtTDP-43 and wtFUS, strains show the presence of insoluble proteins in extracts from whole animals suggesting that protein misfolding may be a primary cause of toxicity. We introduce a genetically tractable platform to investigate motor neuron toxicity caused by mutant TDP-43 and FUS that can be used for suppressor screening.

## Results

### Transgenic worms expressing full-length human TDP-43 or FUS in motor neurons display age-dependent paralysis

**Figure 1:**
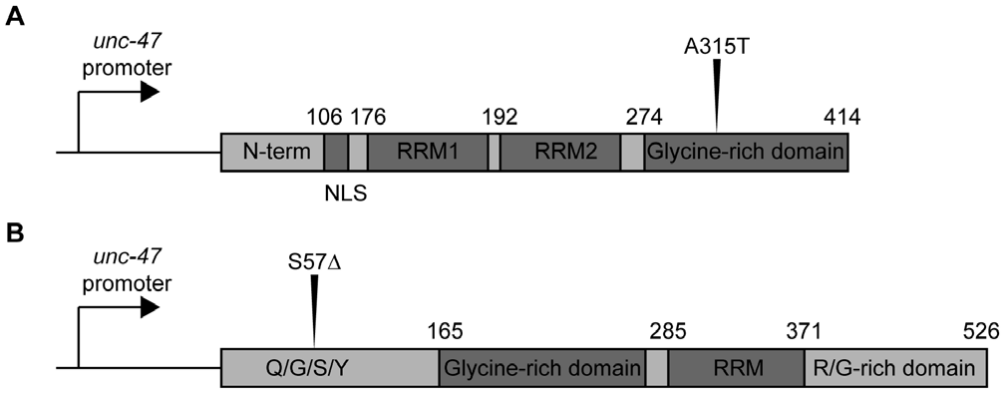
TDP-43 and FUS transgene constructs. (A) Full-length wild type human TDP-43 and the clinical mutation A315T were cloned into a vector for expression in motor neurons by the *unc-47* promoter and injected into *C. elegans*. (B) Full-length wild type human FUS and the clinical mutation S57Δ were cloned into the *unc-47* expression vector and injected into *C. elegans*. RRM (RNA Recognition Motif), Q/G/S/Y (Glutamine-Glycine-Serine-Tyrosine-rich region), R/G (Arginine-Glycine-rich region), NLS (Nuclear localization signal).

**Figure 2:**
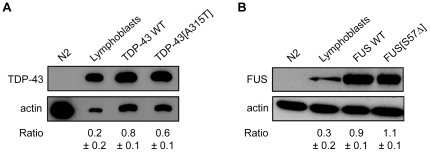
Expression of human TDP-43 and FUS proteins in *C. elegans* transgenics. (A) Total protein levels from non-transgenic worms, human lymphoblast cells and transgenic worms expressing wtTDP-43 or mTDP-43. Staining with a human TDP-43 antibody showed no signal for non-transgenic worms but a signal corresponding to full-length human TDP-43 at ∼45 kDa in size was observed in extracts from human cells and the two transgenic TDP-43 worm strains. wtTDP-43 and mTDP-43 strains showed comparable protein expression levels. (B) Total protein levels from non-transgenic worms, human lymphoblast cells and transgenic worms expressing wtFUS or mFUS. Using a human FUS antibody, no signal was detected in non-transgenic worms, but a signal corresponding to full-length human FUS at ∼75 kDa in size was observed in extracts from lymphoblast cells and the transgenic FUS worm strains. wtFUS and mFUS worms showed identical levels of protein expression. For all experiments actin staining was used as a loading control and expression ratios ± SEM of TDP-43 or FUS to actin was determined from 3 independent experiments. Representative western blots are shown.

Since ALS is a motor neuron disease we expressed wild type and mutant human TDP-43 and FUS proteins in the worm's 26 GABAergic motor neurons with the vesicular GABA transporter (*unc-47*) promoter (Figures 1A, B) [13]. Multiple transgenic strains carrying extrachromosomal arrays were obtained by microinjection and stable lines with chromosomally-integrated transgenes were isolated after UV-irradiation [14]. Both wild type TDP-43 and the ALS-associated A315T mutant proteins were expressed in transgenic worms as detected by immunoblotting of worm protein extracts with a human specific TDP-43 antibody (Figure 2A) [4]. Similarly, using a FUS antibody we confirmed the expression of wild type and the ALS-linked S57Δ mutant proteins by western blotting (Figure 2B) [15].

**Figure 3:**
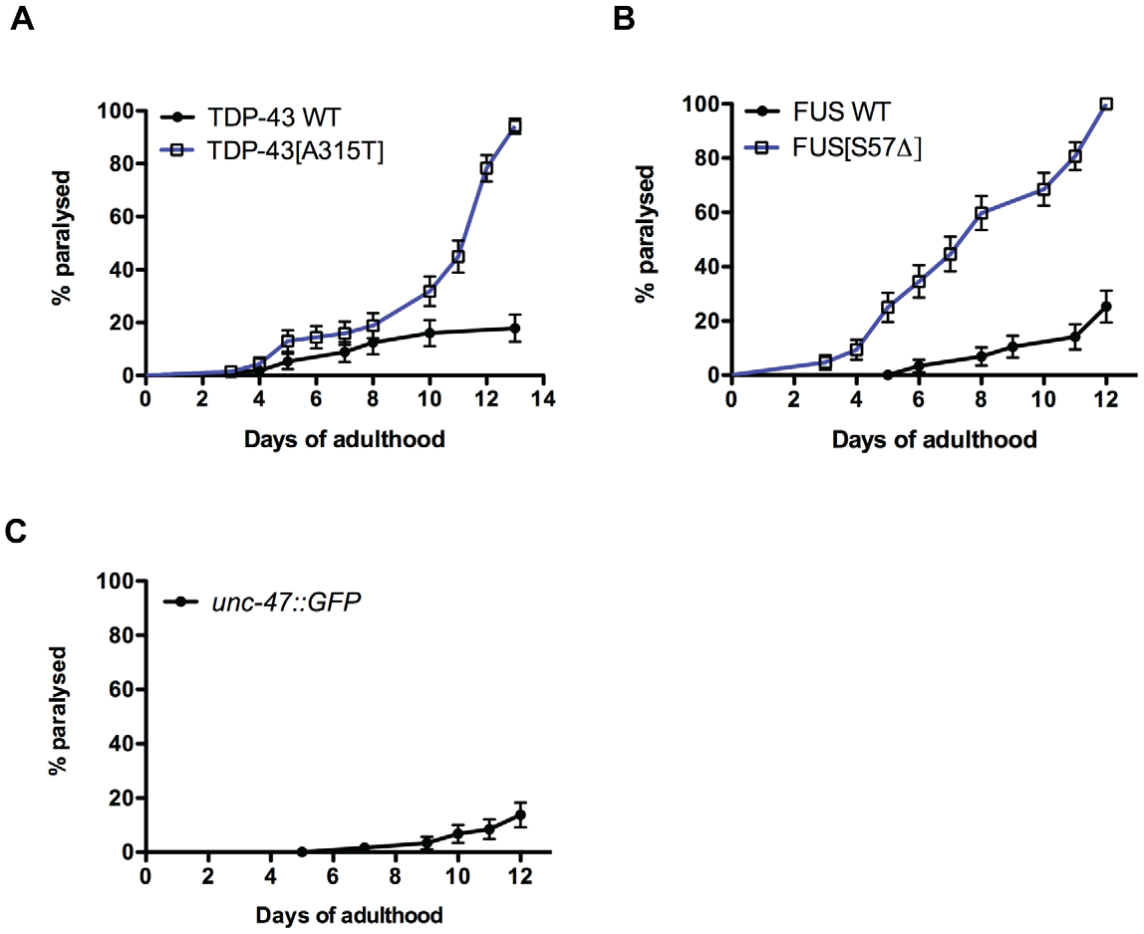
Mutant TDP-43 and FUS cause adult-onset, age-dependent paralysis in *C. elegans*. Transgenics were monitored from the adult stage and scored daily for paralysis. (A) mTDP-43 worms show a rate of progressive paralysis that is greater than transgenics expressing wtTDP-43 (P<0.001). (B) Transgenics expressing mFUS become paralysed significantly sooner than wtFUS control transgenics (P<0.001). (C) Transgenic worms expressing GFP in motor neurons show low levels of paralysis.

All strains were morphologically normal and showed no adverse phenotypes during development. However, during adulthood the transgenic strains begin to display uncoordinated motility phenotypes that progressed to paralysation. Paralysis was age-dependent and occurred at higher rate for mTDP-43 and mFUS worms compared to wtTDP-43 and wtFUS transgenics (Figures 3 A, B). Typically, after 12–13 days on plates 100% of the mTDP-43 and mFUS worms were paralysed while only 20% of the wtTDP-43 and wtFUS worms were affected. The low rate of paralysis for wtTDP-43 and wtFUS strains is comparable to what is observed in transgenics expressing GFP from the same *unc-47* promoter (Figure 3C). Additionally, the paralysis assay is widely used to study age-dependent degenerative phenotypes and is not observed in wild type non-transgenic worms until they reach advanced age (approximately 20 days) [16]–[18]. Finally, motility defects and adult onset paralysis have been previously observed in worms with degenerating GABAergic motor neurons suggesting that mTDP-43 and mFUS may negatively affect GABAergic neuronal function and survival [19].

### TDP-43 and FUS transgenics have normal lifespans

**Figure 4:**
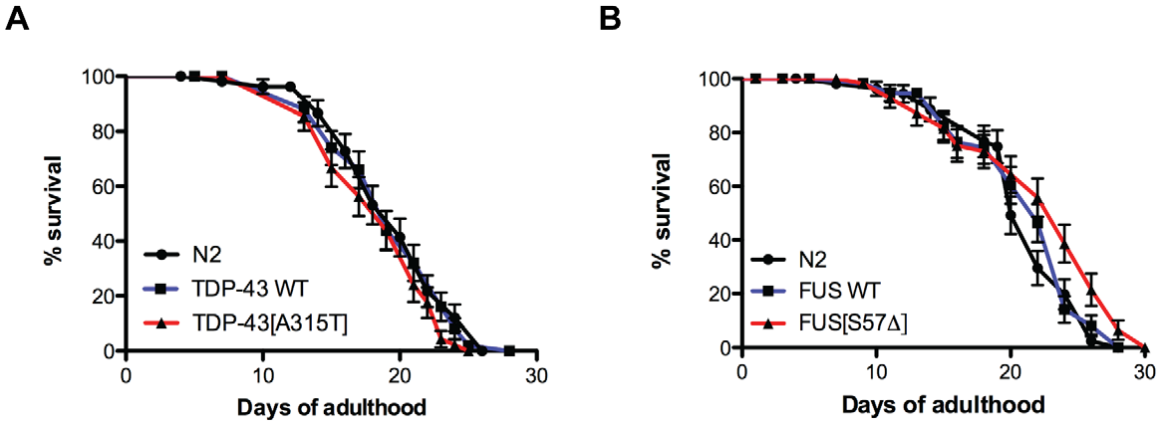
TDP-43 and FUS transgenes do not affect lifespan. Beginning at Day 1 of adulthood we tested the lifespans of wild type non-transgenic N2 worms and transgenics expressing (A) wtTDP-43 and mTDP-43 as well as (B) animals expressing wtFUS and mFUS. Animals expressing TDP-43 or FUS transgenes had lifespans indistinguishable from N2 worms.

One of the signs of aging in worms is decreased motility [18], [20]. Thus the progressive paralysis phenotypes observed in the TDP-43 and FUS transgenics may be due to overall decreased health from the expression of toxic non-native proteins leading to accelerated mortality, a part of which is a decline in motility. We conducted lifespan analyses and observed that all of the transgenics had lifespans indistinguishable from non-transgenic wild type N2 worms (Figures 4A, B and Table S1). These observations suggest that the paralysis observed in our models is specific to the expression of TDP-43 and FUS in motor neurons and not due to secondary effects from general sickness and reduced lifespan.

### TDP-43 and FUS cause neuronal dysfunction

**Figure 5:**
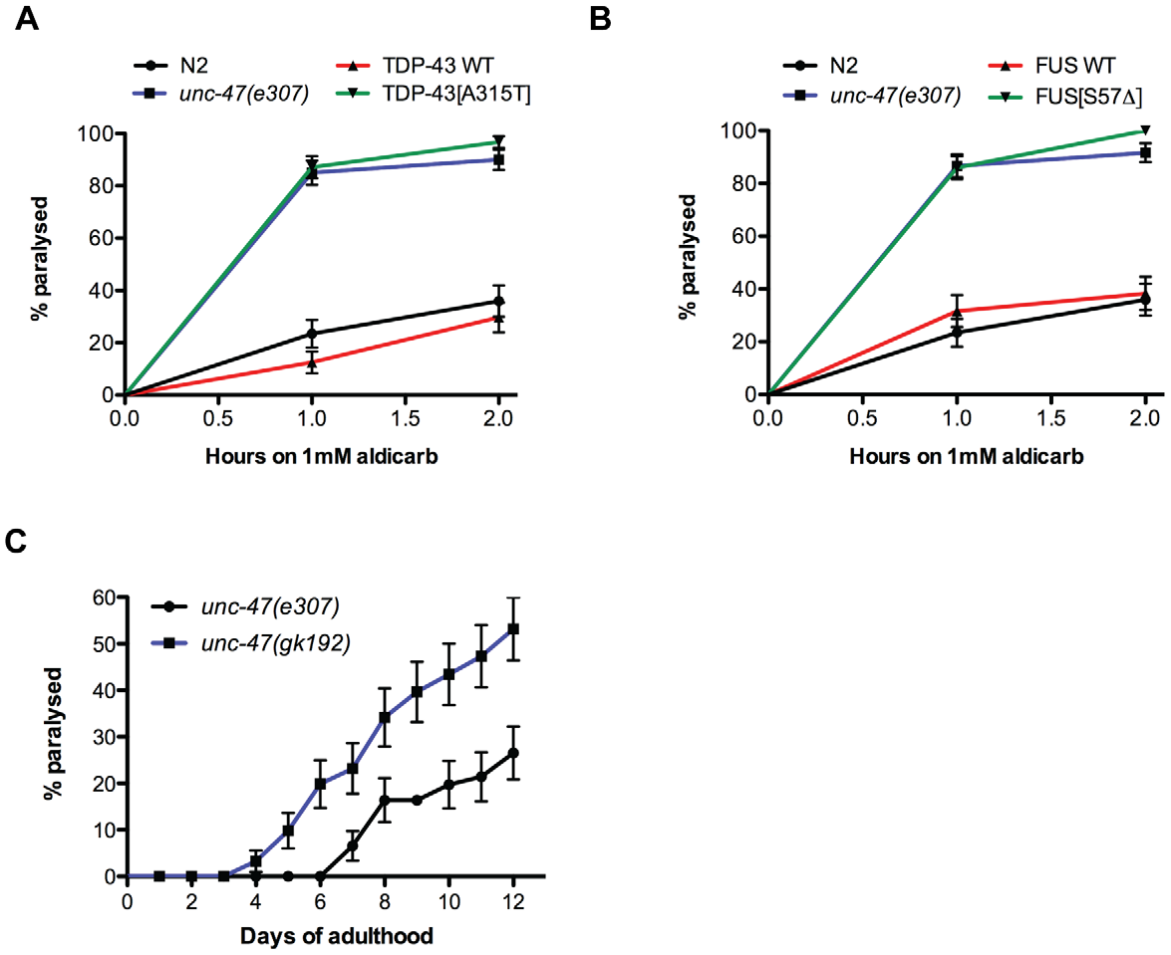
Mutant TDP-43 and FUS impair synaptic transmission. (A) Cholinergic neuronal transmission was measured by determining the onset of paralysis induced by the cholinesterase inhibitor aldicarb. *unc-47(e307)* mutants and mTDP-43 transgenics were hypersensitive to aldicarb-induced paralysis compared to either wtTDP-43 transgenics or N2 worms (P<0.001 for *unc-47* or mTDP-43 compared to N2 or wtTDP-43 worms). (B) mFUS transgenics and *unc-47(e307)* mutants were more sensitive to aldicarb induced paralysis compared to either wtFUS transgenics or N2 controls (P<0.001). (C) *unc-47* mutants grown on regular worm plates showed age-dependent progressive paralysis.

The progressive paralysis phenotypes caused by mTDP-43 and mFUS suggest there may be motor neuron dysfunction and/or degeneration in these animals. *C. elegans* body wall muscle cells receive excitatory (acetylcholine) and inhibitory (GABA) inputs to coordinate muscle contraction/relaxation and facilitate movement [21], [22]. Body wall muscle activity can be measured indirectly with the acetylcholinesterase inhibitor aldicarb [23]. Exposure to aldicarb causes accumulation of acetylcholine at neuromuscular junctions resulting in hyperactive cholinergic synapses, muscle hypercontraction, and acute paralysis [23]. Hypersensitivity to aldicarb-induced paralysis has been used to identify genes that increase acetylcholine secretion or decrease inhibitory GABA signalling [24]. For example mutants lacking genes required for GABA transmission like the vesicular GABA transporter *unc-47* are hypersensitive to aldicarb-induced paralysis [25]. To investigate if our TDP-43 and FUS transgenics had abnormal activity at the neuromuscular junction we exposed the animals to aldicarb. We observed that, like *unc-47* mutants, mTDP-43 and mFUS animals were hypersensitive to aldicarb-induced paralysis, while wtTDP-43 and wtFUS transgenics showed a rate paralysis identical to non-transgenic N2 worms (Figures 5A, B). These data suggest that the inhibitory GABA signalling is impaired in mTDP-43 and mFUS transgenics. *unc-47* mutants are classically described as having a “shrinker” phenotype, where in response to touch the worm does not move away but instead the whole body undergoes longitudinal shortening [21], and we observed that the shrinker phenotype was weakly penetrant in adult mTDP-43 and mFUS worms. To determine if impaired GABAergic neurotransmission contributed to the paralysis phenotype we examined two *unc-47* loss-of-function mutants and they both showed age-dependent paralysis, a phenotype not previously reported for *unc-47* (Figure 5C) [21]. Thus, mTDP-43 and mFUS cause neuronal dysfunction in GABA neurons leading to progressive motility defects culminating in paralysis, a phenotype similar to animals deficient in GABAergic signalling.

### TDP-43 and FUS cause progressive degeneration of motor neurons

**Figure 6:**
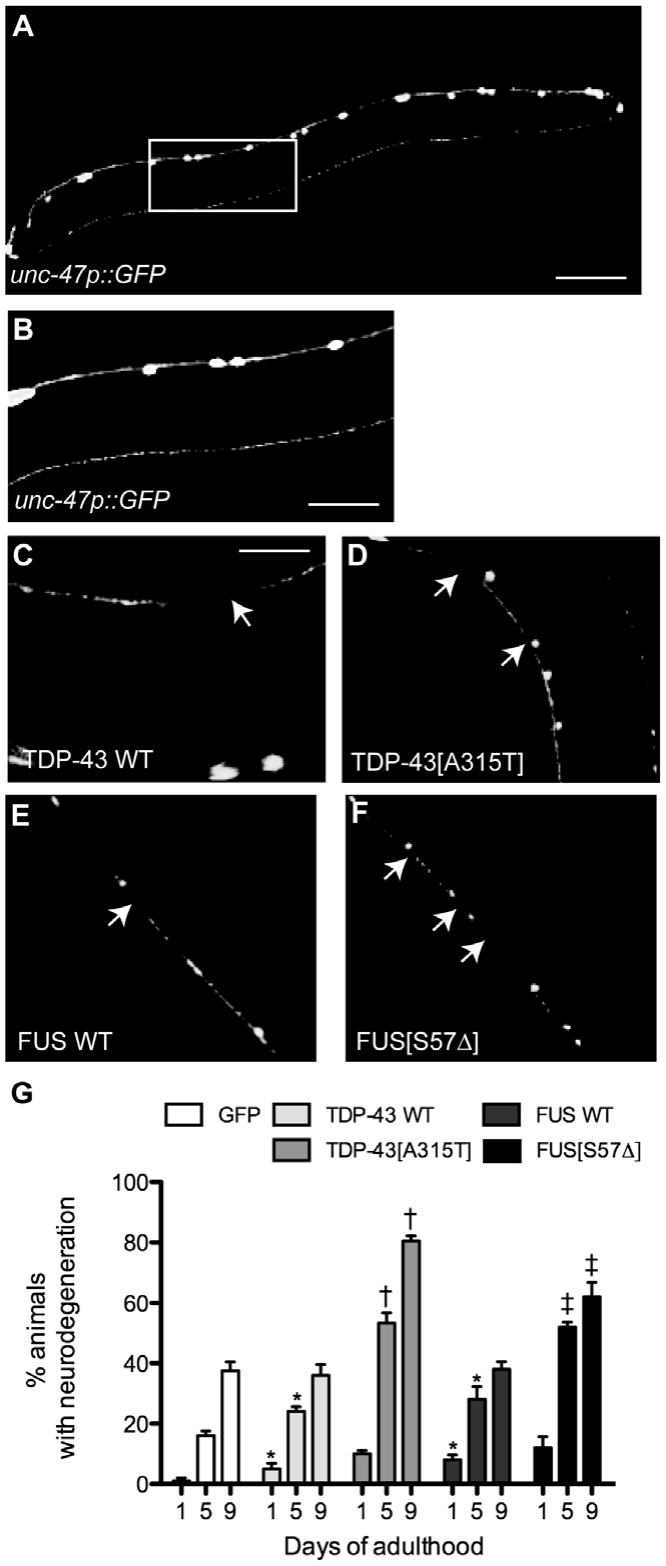
Mutant TDP-43 causes motor neuron degeneration. Shown are representative photos of living, adult *unc-47p::GFP*, *unc-47p::GFP;TDP-43*, and *unc-47p::GFP;FUS* transgenics. (A) Image of an entire *unc-47p::GFP* worm showing the GABAergic motor neurons. Scale bar represents 50 µm. (B) High-magnification of the framed area from (A) showing wild type morphology of motor neurons. Scale bar represents 20 µm. High magnification of motor neurons labelled with *unc-47p::GFP* in (C) wtTDP-43, (D) mTDP-43, (E) wtFUS and (F) mFUS transgenics showing gaps along neuronal processes (arrows). Scale bar represents 10 µm for photos (C) to (F). (G) Quantification of neurodegeneration in transgenic worms at days 1, 5 and 9 of adulthood. * wtTDP-43 and wtFUS have a higher rate of neurodegeneration compared to *unc-47p::GFP* controls at days 1 and 5 of adulthood (P<0.05). †mTDP-43 transgenics have a higher rate of neurodegeneration at days 5 and 9 compared to wtTDP-43 transgenics (P<0.001). ‡mFUS transgenics show an enhanced rate of neurodegeneration at days 5 and 9 of adulthood in compared to wtFUS transgenics (P<0.001).

Many neurodegenerative diseases are characterized by neuronal dysfunction prior to degeneration [26]. To investigate if the progressive paralysis phenotypes in our TDP-43 and FUS transgenics were accompanied by neurodegeneration we crossed all of the transgenics with an integrated reporter (*unc-47p::GFP*) that expresses GFP in the same GABAergic motor neurons [13] (Figures 6A, B). Similar to reports from another *C. elegans* TDP-43 toxicity model [27], we observed gaps/breaks in motor neuron processes in TDP-43 and FUS animals compared to animals expressing *unc-47p::GFP* alone (Figures 6 C–F). We extended our analysis by scoring degeneration in living GFP, wtTDP-43, mTDP-43, wtFUS and mFUS transgenics at days 1, 5 and 9 of adulthood. We observed that degeneration was age-dependent and occurred at higher rate for the mTDP-43 and mFUS animals compared to the wtTDP-43 and wtFUS transgenics (Figure 6G). Thus our TDP-43 and FUS transgenics mimic the adult-onset, gradual decline of neuronal function ultimately resulting in age-dependent motor neuron degeneration seen in diseases like ALS.

### Mutant TDP-43 and FUS are highly insoluble

**Figure 7:**
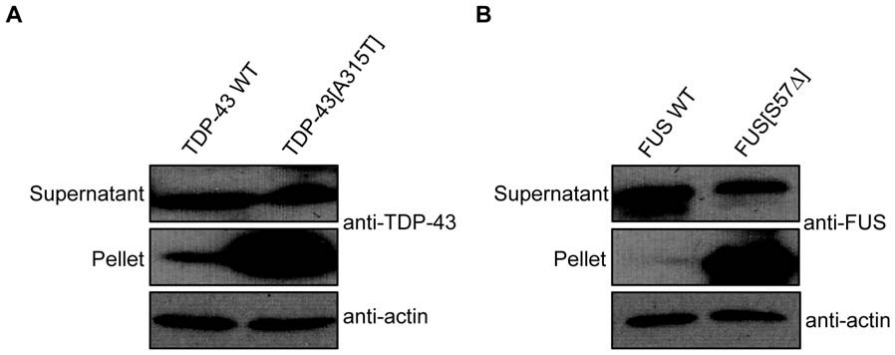
Mutant TDP-43 and FUS are highly insoluble. Shown are representative images from western blotting of the soluble supernatant and insoluble pellet fractions of protein extracts from transgenic TDP-43 and FUS strains. (A) Blotting against TDP-43 shows that a large proportion of the TDP-43 signal resides in the insoluble fraction for mTDP-43 worms, while the signal is largely soluble for the wtTDP-43 samples. (B) Immunoblotting with a human FUS antibody revealed that mFUS proteins primarily resided in the insoluble fractions while wtFUS proteins were exclusively soluble. Immunoblotting for actin was used as the loading control.

Since TDP-43 and FUS are prone to aggregation in several model systems including *C. elegans*, we tested if the same was true for our transgenics [27]–[33]. To examine if protein misfolding is more pronounced for strains expressing mTDP-43 and mFUS, we used a biochemical assay to detect protein aggregation. Homogenized protein extracts from transgenic worms were separated into supernatant (detergent-soluble) and pellet (detergent-insoluble) fractions [30]. Immunoblotting the TDP-43 transgenics with a human TDP-43 antibody revealed the accumulation of mTDP-43 in the pelleted, insoluble fraction, while wtTDP-43 proteins were predominantly detected in the supernatant, or soluble fractions (Figure 7A). Similar results were obtained for the FUS transgenics where immunoblotting with a human FUS antibody showed that mFUS accumulated in the insoluble pellet fraction while wtFUS proteins remained soluble (Figure 7B). These data suggest that mTDP-43 and mFUS proteins are susceptible to misfolding leading to insolubility and aggregation that may contribute to motor neuron dysfunction and degeneration.

**Figure 8:**
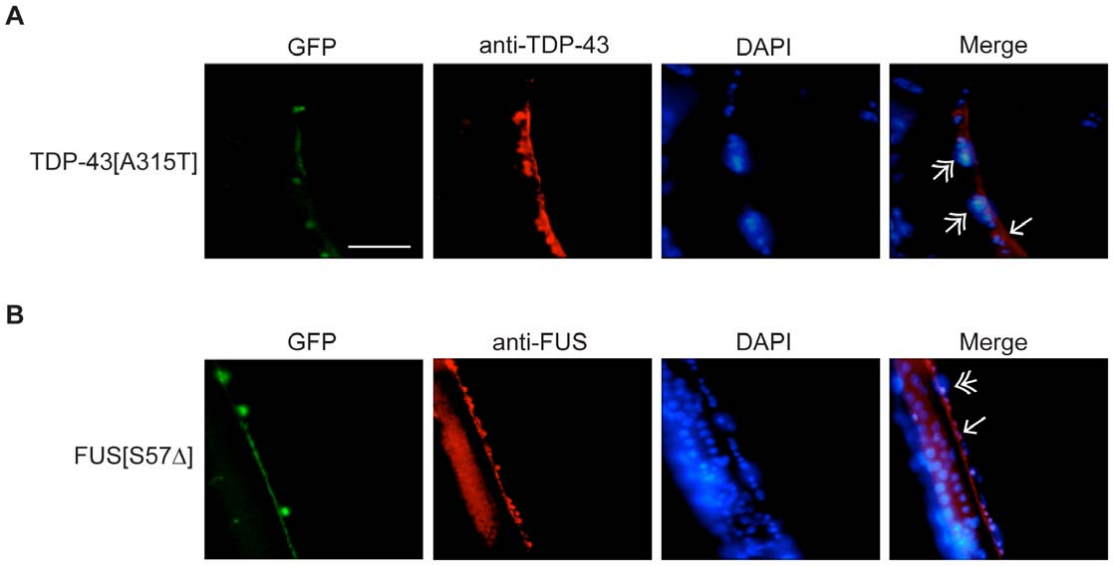
Mutant TDP-43 and FUS aggregate in vivo. (A) Representative image of a fixed *unc-47p::GFP;mTDP-43* worm stained with a human TDP-43 antibody. The green channel shows GFP labelled motor neurons. Antibody staining (red signal) revealed aggregation of TDP-43 signals in motor neurons. Staining of motor neuron nuclei with DAPI (blue signal) revealed that TDP-43 is both cytoplasmic (single arrowhead) and nuclear (double arrowhead). Scale bar represents 10 µm for all photos. (B) Staining of *unc-47p::GFP;mFUS* worms with a human FUS antibody (red signal) and DAPI (blue signal) revealed cytoplasmic (single arrowhead) and nuclear (double arrowhead) accumulations in motor neurons.

Next focusing on the mTDP-43 and mFUS transgenics we fixed whole *unc-47p::GFP;mTDP-43* and *unc-47p::GFP;mFUS* worms and respectively stained them with human TDP-43 and human FUS antibodies. We detected mTDP-43 and mFUS in both the nuclei and cytoplasm of motor neurons (Figure 8). The cytoplasmic accumulation of mTDP-43 and mFUS in our transgenics is consistent with findings in patients suggesting that these proteins misfold leading to intracellular build-up and aggregation [10].

**Figure 9:**
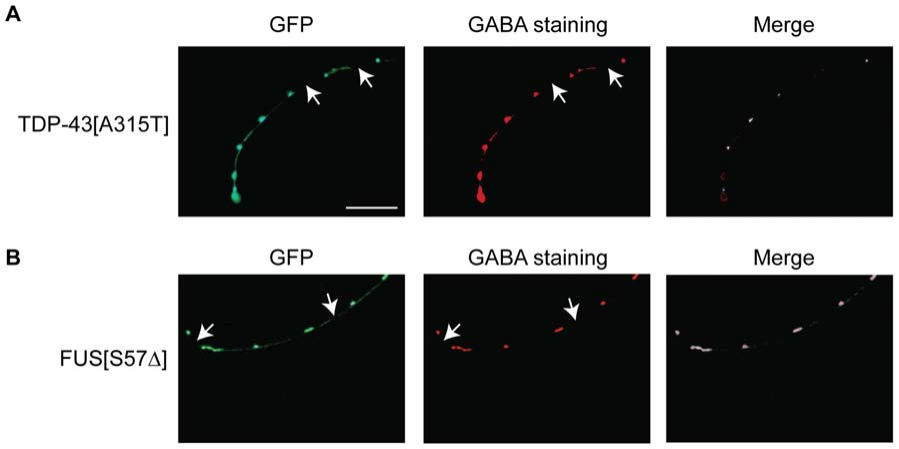
Decreased GABA staining in mutant TDP-43 and FUS worms. (A) Fluorescent micrograph of a fixed *unc-47p::GFP;mTDP-43* worm stained with a GABA antibody revealed neurodegeneration in motor neurons that mirrors the loss of GFP signals. Scale bar represents 10 µm for all photos. (B) Staining of *unc-47p::GFP;mFUS* worms also showed loss of GABA signals similar to the loss of GFP in the motor neurons.

Finally, we noticed that the fixed mTDP-43 and mFUS showed gaps or breaks along the GFP labelled neuronal processes similar to what was observed in living animals (Figures 6D, F). To confirm that neurodegeneration was not simply due to loss of GFP signals, we stained whole *unc-47p::GFP;mTDP-43* and *unc-47p::GFP;mFUS* worms for GABA [22]. We observed that the gaps along the processes as visualized by a loss of GFP signal likewise corresponded to a loss of GABA staining (Figure 9). Altogether these data suggest that the expression of TDP-43 and FUS lead to degeneration of motor neurons as has been observed for TDP-43 in other worm models [27].

### Paralysis phenotypes are enhanced in liquid culture

One goal in developing these transgenics is for use in genetic and pharmacological suppressor screens. TDP-43 and FUS transgenics may have decreased inhibitory GABA signalling ultimately causing muscle hypercontraction leading to paralysis. When grown on solid media the mTDP-43 and mFUS paralysis phenotypes manifest over a period of 5 to 13 days (Figure 3). Worms grown in liquid culture exhibit a stereotypical swimming motion that is considerably more vigorous than worms crawling on solid media [34]. We hypothesized that placing worms in liquid culture would increase activity at the neuromuscular junction and precipitate paralysis phenotypes much earlier than worms grown on solid media.

**Figure 10:**
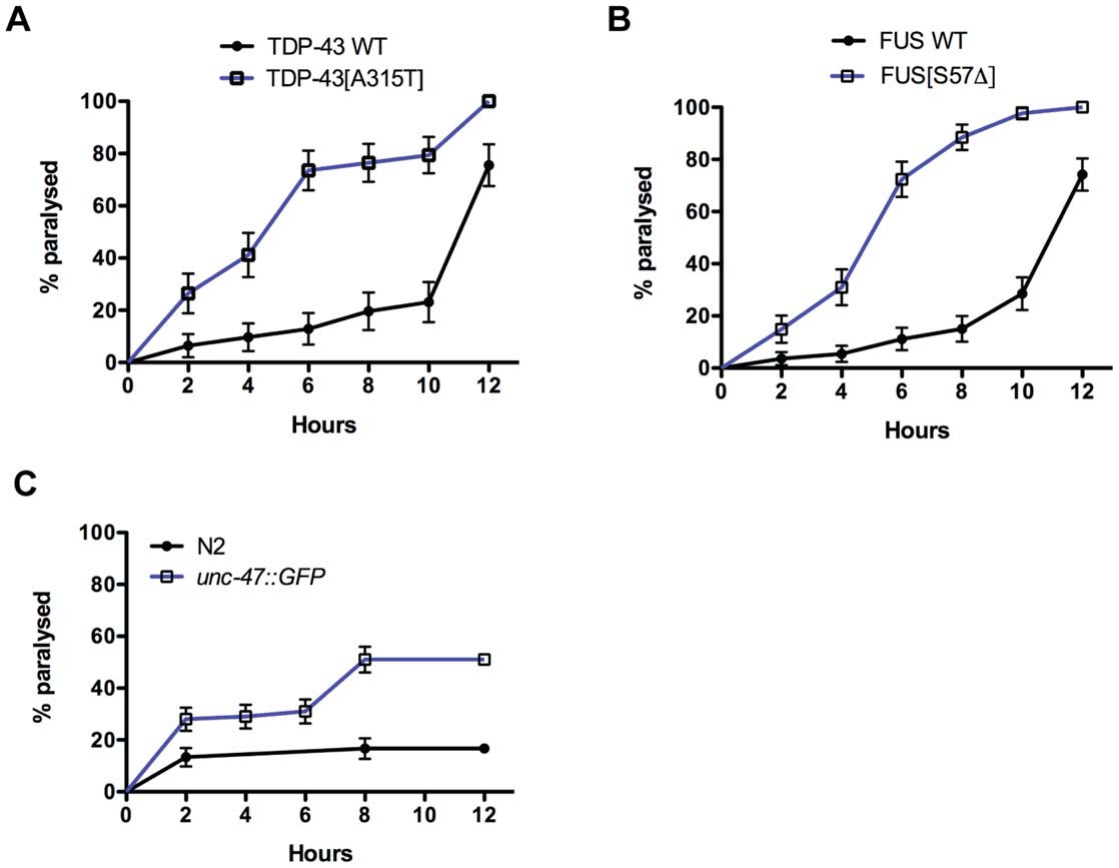
Accelerated paralysis phenotypes for TDP-43 and FUS transgenics in liquid culture. (A) Paralysis phenotypes resolve over a number of hours for wtTDP-43 and mTDP-43 worms grown in liquid culture. mTDP-43 worms have a faster rate of paralysis compared to wtTDP-43 transgenics (P<0.001). (B) Transgenic mFUS worms show motility defects and become paralysed at a rate faster than wtFUS controls (P<0.001). (C) *unc-47p::GFP* transgenics have an increased rate of paralysis compared to non-transgenic N2 worms (P<0.001).

Using age-synchronized worms we transferred young adult TDP-43 and FUS transgenics to 96-well plates with liquid media and scored their motility every 2 hours. We observed a rapid onset of paralysis for the mTDP-43 and mFUS lines with approximately 80% of the population becoming immobile after 6 hours progressing to 100% paralysis after 12 hours (Figure 10A, B Videos S1, S2, S3, S4). wtTDP-43 and wtFUS animals also showed increased paralysis but at a much lower rate, with approximately 20% of the animals immobile after 6 hours moving to 80% paralysis after 12 hours (Figure 10, Videos S5, S6, S7, S8). Non-transgenic N2 animals showed a very low rate of paralysis of approximately 15% after 12 hours (Figure 10C, Videos S9, S10). In comparison, approximately 50% of transgenic *unc-47p::GFP* control animals were paralysed after 12 hours, a rate intermediate between non-transgenic N2 worms and transgenic wtTDP-43 and wtFUS animals (Figure 10C, Videos S11, S12). The difference between wild type and mutant transgenic lines is easy to distinguish, particularly at 6 hours, and suggests that this phenotype may be used for rapid genetic and chemical screening.

## Discussion

Here we introduce a novel *C. elegans* platform for investigating mechanisms of motor neuron toxicity caused by mTDP-43 and mFUS. To more closely model human disease we chose to express full-length human TDP-43 and FUS without additional tags since the inclusion of tags like GFP can mask or enhance the phenotypes of wild type and mutant proteins [35], [36]. Additionally, we reasoned that restricting expression to a smaller set of neurons might produce phenotypes less severe, or later, than observed in other *C. elegans* models [27], [29], [30], [33]. Since ALS is characterized by degeneration of the motor neurons we engineered strains expressing human TDP-43 and FUS in the animal's 26 GABAergic neurons [13], [22]. Additionally, ALS patients show cortical hyperexcitability that may be due to reduced inhibitory signalling from the GABAergic system [37], [38]. We believe our transgenic mTDP-43 and mFUS worms recapitulate this pathophysiological mechanism; they show decreased GABA staining and are hypersensitive to the acetylcholinesterase inhibitor aldicarb, suggesting a reduction of inhibitory GABA input at neuromuscular junctions [24], [25]. In our models sensitivity to aldicarb can be detected in day 1 adult worms, while paralysis and motor neuron degeneration can first be detected starting at day 5 of adulthood demonstrating that similar to ALS, neuronal dysfunction occurs prior to neurodegeneration [39].

Importantly, our transgenic TDP-43 and FUS animals only begin to show motility defects once they have reached adulthood a feature absent from other models [27], [29], [30], [33]. Thus our models mirror a prominent clinical feature of ALS, they display adult-onset, age-dependent, progressive paralysis [40], [41]. Additionally, unlike previously described TDP-43 and FUS models based on pan-neuronal expression [27], [30], [33] our transgenics do not show reduced lifespan suggesting the behavioural phenotypes observed in our transgenics are not influenced by general sickness. Our transgenics do share many features with other neuronal-based models, notably the aggregation and insolubility of mutant TDP-43 and FUS as well as degeneration of motor neurons suggesting there may be common mechanisms of toxicity amongst the models [27], [29], [30], [32], [33], [42]–[45]. However, cytoplasmic aggregation of TDP-43 and FUS is a prominent feature of the human pathologies and this is seen in a recently described worm FUS model [33], but is absent from previously reported TDP-43 models [27], [29], [30]. We detect TDP-43 and FUS in both the nucleus and the cytoplasm of motor neurons from young adult (Day 1) transgenics. The preferential toxicity of mutant TDP-43 and FUS alleles along with their cytoplasmic accumulation suggests our models may recapitulate aspects of neurotoxicity relevant to the disease state.

With no clear mechanism for TDP-43 and FUS neuronal toxicity it is currently not possible to design *in vitro* assays for high-throughput drug screening. Thus the further development and characterization of *in vivo* models for neurodegeneration will guide studies in mammalian systems. We believe our models strike an optimal balance between strong, age-dependent phenotypes and the expression of mutant proteins in relatively few neurons and may be useful for modifier screening. In terms of sensitivity, genetic mechanisms and/or small molecules need only to work on 26 neurons to achieve suppression. In terms of speed, our transgenics offer the possibility of medium-throughput suppressor screening based on the accelerated paralysis phenotype of mTDP-43 and mFUS worms grown in liquid culture. mTDP-43 and mFUS cause neuronal dysfunction in advance of motor neuron degeneration. The path from protein misfolding to neuronal dysfunction and cell death takes many decades in humans and it may be more efficient to target therapies to early pathogenic stages. Thus using simple systems to screen for suppression of neuronal dysfunction may be useful to prevent subsequent neurodegeneration.

A number of models for TDP-43 and FUS toxicity in various systems have been described, but there is still no clear answer whether TDP-43 and FUS neuronal toxicity are due to a loss/gain of function of these proteins individually or together in some common genetic pathway [44]–[46]. Furthermore it is still unclear if all TDP-43 and FUS mutations share similar pathogenic mechanisms but having similarly constructed models for each may address this question. Now that we have validated the *unc-47* motor neuron approach for modelling toxicity, future work will focus on the development of new transgenics with additional TDP-43 and FUS mutations.

We present here novel transgenics for investigating age-dependent motor neuron toxicity caused by mutant TDP-43 and FUS. We expect these strains will be useful for identifying genetic and chemical suppressors to give insights into disease mechanisms and support the development of new therapies for age-dependent neurodegeneration.

## Materials and Methods

### Nematode strains

Standard methods of culturing and handling worms were used [47]. Worms were maintained on standard NGM plates streaked with OP50 *E. coli*. Strains used in this study were obtained from the *C. elegans* Genetics Center (University of Minnesota, Minneapolis) and include: N2, *oxIs12[unc-47p::GFP+lin-15]*, *unc-47(e307)*, *unc-47(gk192)* and *unc-119(ed3)*.

### Transgenic TDP-43 and FUS worms

Human cDNAs for wild type and mutant TDP-43[A315T], and wild type and mutant FUS-TLS[S57Δ] were obtained from Dr. Guy Rouleau (CRCHUM, Université de Montréal). The cDNAs were amplified by PCR and cloned into the Gateway vector pDONR221 following the manufacturer's protocol (Invitrogen). Multisite Gateway recombination was performed with the pDONR TDP-43 and FUS clones along with clones containing the *unc-47* promoter (kind gift from Dr. Erik Jorgensen, University of Utah), the *unc-54* 3′UTR plasmid pCM5.37 (Dr. Geraldine Seydoux, Johns Hopkins, Addgene plasmid 17253) and the destination vector pCFJ150 to create *unc-47*::TDP-43 and *unc-47*::FUS expression vectors. Transgenic lines were created by microinjection of *unc-119(ed3)* worms, multiple lines were generated and strains behaving similarly were kept for further analysis. Transgenes were integrated by UV irradiation and lines were outcrossed to wild type N2 worms 5 times before use. The main strains used in this study include: *xqIs132[unc-47::TDP-43-WT;unc-119(+)]*, *xqIs133[unc-47::TDP-43[A315T];unc-119(+)]*, *xqIs173[unc-47::FUS-WT;unc-119(+)]*, and *xqIs98[unc-47::FUS[S57Δ];unc-119(+)]*.

### Paralysis assays on plates

For worms expressing TDP-43 or FUS, 20–30 adult day 1 animals were picked to NGM plates and scored daily for movement. Animals were counted as paralyzed if they failed to move upon prodding with a worm pick. Worms were scored as dead if they failed to move their head after being prodded in the nose and showed no pharyngeal pumping. All experiments were conducted at 20°C.

### Lifespan assays

Worms were grown on NGM-FUDR plates to prevent progeny from hatching. 20 animals/plate by triplicates were tested at 20°C from adult day 1 until death. Worms were declared dead if they did not respond to tactile or heat stimulus. Survival curves were produced and compared using the Log-rank (Mantel-Cox) test.

### Aldicarb test

To evaluate synaptic transmission, worms were grown on NGM and transferred to NGM plates +1 mM aldicarb at adult day 1. Paralysis was scored after 1 and 2 hours on aldicarb plates. Animals were counted as paralyzed if they failed to move upon prodding with a worm pick. All tests were performed at 20°C.

### Liquid culture protocol

Synchronized populations of worms were obtained by hypochlorite extraction. Young adult worms were distributed in 96-wells plate (20 µl per well; 20–30 worms per well), containing DMSO or test compounds and incubated for up to 6 h at 20°C on a shaker. The motility test was assessed by stereomicroscopy. Videos of worms were taken with on an Olympus S7x7 stereomicroscope equipped with a Grasshopper GRAS-03K2M camera using Flycap software (Point Grey Research) at a rate of 300 frames per second.

### Immunostaining of whole worms

Age synchronized, adult day 1, whole worms were fixed and stained as described in WormBook [48]. Antibodies used include: rabbit anti-TDP-43 (1∶50, Proteintech), rabbit anti-FUS/TLS (1∶50, AbCam), and rabbit anti-GABA (1∶50, Proteintech).

### Fluorescence microscopy

For scoring gaps/breaks from TDP-43 and FUS transgenics, synchronized animals were selected at days 1, 5 and 9 of adulthood for visualization of motor neurons *in vivo*. Animals were immobilized in M9 with 5 *mM* levamisole and mounted on slides with 2% agarose pads. Motor neurons were visualized with a Leica 6000 microscope and a Leica DFC 480 camera. A minimum of 100 animals was scored per treatment over 4–6 trials. The mean and SEM were calculated for each trial and two-tailed t-tests were used for statistical analysis.

### Worm lysates

Worms were collected in M9 buffer, washed 3 times with M9 and pellets were placed at −80°C overnight. Pellets were lysed in RIPA buffer (150 mM NaCl, 50 mM Tris pH 7.4, 1% Triton X-100, 0.1% SDS, 1% sodium deoxycholate)+0.1% protease inhibitors (10 mg/ml leupeptin, 10 mg/ml pepstatin A, 10 mg/ml chymostatin LPC; 1/1000). Pellets were passed through a 27_1/2_ G syringe 10 times, sonicated and centrifuged at 16000*g*. Supernatants were collected.

### Protein quantification

All supernatants were quantified with the BCA Protein Assay Kit (Thermo Scientific) following the manufacturer instructions.

### Protein solubility

For TDP-43 and FUS transgenics soluble/insoluble fractions, worms were lysed in Extraction Buffer (1 M Tris-HCl pH 8, 0.5 M EDTA, 1 M NaCl, 10% NP40+protease inhibitors (LPC; 1/1000)). Pellets were passed through a 27_1/2_ G syringe 10 times, sonicated and centrifuged at 100000*g* for 5 min. The soluble supernatant was saved and the remaining pellet was resuspended in extraction buffer, sonicated and centrifuged at 100000g for 5 min. The remaining pellet was resuspended into 100 µl of RIPA buffer, sonicated until the pellet was resuspended in solution and saved.

### Immunoblots

Worm RIPA samples (175 µg/well) were resuspended directly in 1× Laemmli sample buffer, migrated in 12.5% or 10% polyacrylamide gels, transferred to nitrocellulose membranes (BioRad) and immunoblotted. Antibodies used: rabbit anti-human-TDP-43 (1∶200, Proteintech), rabbit anti-human-FUS/TLS (1∶200, AbCam), and mouse anti-actin (1∶10000, MP Biomedical). Blots were visualized with peroxidase-conjugated secondary antibodies and ECL Western Blotting Substrate (Thermo Scientific). Densitometry was performed with Photoshop (Adobe).

### Statistics

For paralysis and stress-resistance tests, survival curves were generated and compared using the Log-rank (Mantel-Cox) test, and 60–100 animals were tested per genotype and repeated at least three times. For image analysis statistical significance was determined by Student's t-test and the results shown as mean ± standard error. Prism 5 (GraphPad Software) was used for all statistical analyses.

## Supporting Information

Table S1 **Lifespan analysis for all experiments.**
(PDF)

Video S1 **mTDP-43 worms in liquid culture at time 0.**
(MOV)

Video S2 **mTDP-43 worms after 6 hours in liquid culture.**
(MOV)

Video S3 **mFUS worms in liquid culture at time 0.**
(MOV)

Video S4 **mFUS worms after 6 hours in liquid culture.**
(MOV)

Video S5 **wtTDP-43 worms in liquid culture at time 0.**
(MOV)

Video S6 **wtTDP-43 worms after 6 hours in liquid culture.**
(MOV)

Video S7 **wtFUS worms in liquid culture at time 0.**
(MOV)

Video S8 **wtFUS worms after 6 hours in liquid culture.**
(MOV)

Video S9 **N2 worms in liquid culture at time 0.**
(MOV)

Video S10 **N2 worms after 6 hours in liquid culture.**
(MOV)

Video S11 ***unc-47p::GFP*** **worms in liquid culture at time 0.**
(MOV)

Video S12 ***unc-47p::GFP*** **worms after 6 hours in liquid culture.**
(MOV)
