# Supplementary material for: Mutant TDP-43 and FUS Cause Age-Dependent Paralysis and Neurodegeneration in C. elegans
Source: PLoS One. 2012 Feb 21;7(2):e31321. doi: 10.1371/journal.pone.0031321 (PMC3283630; doi:10.1371/journal.pone.0031321)
Supplement: Table S1 — Lifespan analysis for all experiments. (PDF) [file pone.0031321.s001.pdf]

Tables

	Strain	Mean Lifespan	P value	75th Percentile (Days)	Maximum Lifespan	Total Number of Animals Died/Total
**Figure 4**	N2	18		22	26	98/112
	TDP-43 WT	18	ns 0.6367	22	28	100/104
	TDP-43[A315T]	18	ns 0.0534	24	25	94/102
	N2	20		23	28	98/112
	FUS WT	21	ns 0.4172	24	28	100/122
	FUS[S57Δ]	22	ns 0.0890	24	30	96/124

Table S1, related to Figure 4. Lifespan analysis for all experiments.

Animals that died prematurely (ruptured, internal hatching) or were lost (crawled off the plate) were censored at the time of scoring. All control and experimental animals were scored and transferred to new plates at the same time. ns: not significant
